# Biocompatible Supramolecular Mesoporous Silica Nanoparticles as the Next-Generation Drug Delivery System

**DOI:** 10.3389/fphar.2022.886981

**Published:** 2022-06-28

**Authors:** Farahidah Mohamed, May K. Oo, Bappaditya Chatterjee, Batoul Alallam

**Affiliations:** ^1^ Pharmaceutical Technology Department, Kulliyyah of Pharmacy, International Islamic University Malaysia, Kuantan, Malaysia; ^2^ Shobhaben Pratapbhai Patel School of Pharmacy & Technology Management, SVKM’s Narsee Monjee Institute of Management Studies, Mumbai, India; ^3^ Cluster of Integrative Medicine, Advanced Medical and Dental Institute, University of Science Malaysia, Penang, Malaysia

**Keywords:** mesoporous, silica, nanoparticles, insulin, pharmaceutical, oral

## Abstract

Supramolecular mesoporous silica nanoparticles (MSNs) offer distinct properties as opposed to micron-sized silica particles in terms of their crystal structure, morphology–porosity, toxicity, biological effects, and others. MSN biocompatibility has touched the pharmaceutical realm to exploit its robust synthesis pathway for delivery of various therapeutic molecules including macromolecules and small-molecule drugs. This article provides a brief review of MSN history followed by special emphasis on the influencing factors affecting morphology–porosity characteristics. Its applications as the next-generation drug delivery system (NGDDS) particularly in a controlled release dosage form via an oral drug delivery system are also presented and shall be highlighted as oral delivery is the most convenient route of drug administration with the economical cost of development through to scale-up for clinical trials and market launch.

## Introduction

The development of a new oral delivery system for small molecular weights and biologics could bring significant benefits to suffering patients due to its simplicity of the method of administration which can avoid the pain compared to the regular injection methods. Mesoporous silica (MPS) particles coupled with enteric coating polymer are also possible and like “dual-technology” working along together to provide the desired release profile. This mini-review will provide a discussion on the history and potential use as next-generation drug delivery system of mesoporous silica nanoparticles, especially in modified-release strategies.

## History of Mesoporous Silica Nanoparticles

The prelude of mesoporous development dated back to the 1990s with the first synthesis of mesoporous silica materials from aluminosilicate gels. The research team from Mobil Research and Development Corporation, located in Paulsboro, New Jersey (United States), accomplished the synthesis by employing the templating mechanism of the silica liquid crystal and cationic surfactants in 1992 (Narayan et al., 2018). Thereafter, the mesoporous inventions with various geometrical shapes such as thin film (Pevzner et al., 1999) and cylindrical have been known as “MCM” to represent Mobil Crystalline Materials or Mobil Composition of Matter. About 3 years gap, another invention of mesoporous silica was attempted by researchers in Japan (Inagaki et al., 1996). This time a layered polysilicate kanemite was used as the template with quaternary ammonium surfactant to form folded sheets with a highly ordered mesoporous structure which was thereafter named folded sheets mesoporous (FSM) (Inagaki et al., 1996). The syntheses were reported to take place best initially in high pH ∼ 11 to allow effective cation exchange and subsequently readjusted to pH 8.5 for the condensation process.

Another venture had seen researchers from the University of Santa Barbara, California, demonstrated the first highly ordered amorphous silica structure in 1998 by testing various surface-directing agents from non-ionic and low molecular weight surfactants including sorbitan-based Tween® series, Brij™, and Pluronic® series with a diluted aqueous organic concentration media (Zhao et al., 1998). They found that the best working pH was approximately below 2 which was the isoelectric point of cationic silica to consistently yield highly ordered silica species using tetraethyl orthosilicate or tetraethoxysilane (TEOS) as silica precursor. Subsequently, the mesoporous from this family has been called ‘Santa Barbara Amorphous’ coded with “SBA.” In 2000, SBA-based mesoporous were further evolved by substitution of relatively more expensive TEOS with inexpensive sodium metasilicate as the source of silica (Kim and Stucky, 2000) yielding cubic MSN.

In 2001, a new templating method was developed by the Technology University of Delft (denoted as TUD), Netherlands, without the need to employ any surfactants. Three main ingredients for this method were water, silica source (i.e. TEOS), and inexpensive water-miscible organic material such as triethanolamine which is commonly used as a buffer. These materials were subjected to homogenous mixing, followed by aging, solidification, and calcination yielding foam-like mesoporous silica nanoparticles.

Additionally, the research team from the Hiroshima University of Japan had exploited the effects of polymerizing styrene molecules to polystyrene during the synthesis of mesoporous silica in 2009 (Nandiyanto et al., 2009). The synthesis process started with a water-in-oil emulsion composed of cationic surfactant to stabilize the emulsion while behaving as a template for the silica nanoclusters to grow as well as the polymerization of styrene. Both molecules self-assembled inside the micelles of which the surfactants and the polystyrene were finally removed leaving the mesoporous silica in a spherical shape. Such silica nanoparticles were, hence, named as Hiroshima Mesoporous Material (HMM).

As mentioned earlier, we can see the invention centered around porous silica is highly robust and competitive. Of particular importance is the synthesis of nanoporous silica materials which is customizable in terms of the pore size, shape, composition, and spatial nano-environment within the matrix with numerous possible chemical reactions, including silanol functionalization. Porous materials can generally be classified as macroporous (>50 nm), mesoporous (2–50 nm), and microporous materials (<2 nm). Mesoporous silica range provides high specific spatial areas and large pore volume which are of technological importance for numerous possibilities of inclusion with therapeutic molecules. The possible size for the latter is estimated to be around 1 to 0.6 nm (Heikkil et al., 2007) to ensure it fits the confined pores. Without the need for silanol functionalization, any molecule with an acidic functional group like ibuprofen is anticipated to be encapsulated well within the mesoporous matrix.

The particle size of MSN can also be engineered as required by altering the pH of the synthesis medium or by the addition of size-effecting co-template like glycerol (Wang et al., 2009; Johansson et al., 2011). Varying particle size or aspect ratios of the particles affected cellular uptake (Hao et al., 2012), internalization rates, and various cellular function including cell proliferation, cytoskeleton formation, migration, adhesion, and apoptosis (Xinglu et al., 2010; Lin et al., 2018). These also suggested that larger particles demonstrated a slower initial *in vitro* drug release followed by a more rapid drug release compared to the smaller particles with rapid burst release followed by gradual release.

**FIGURE 1:**
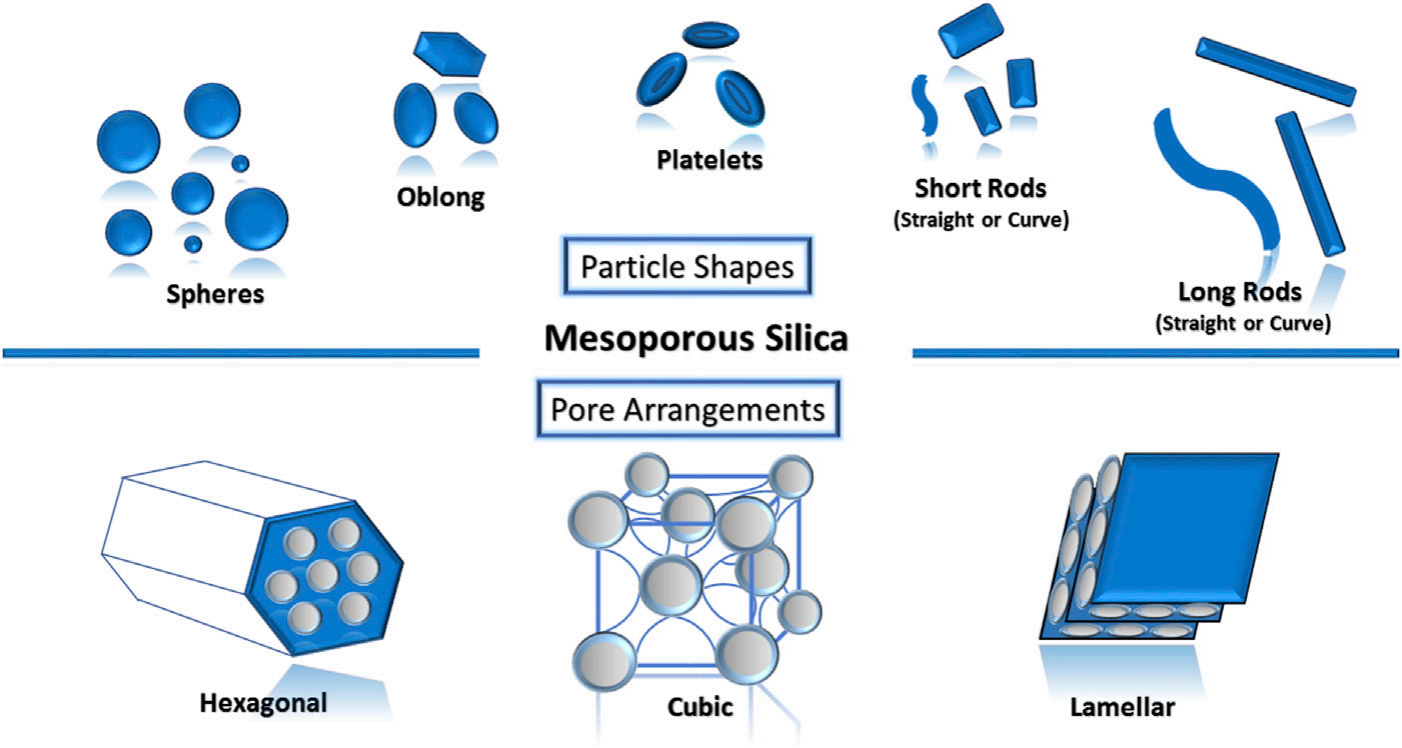
Various types of mesoporous silica nanoparticles can be designed based on the intended purpose.

The role of surfactants to modulate porosity–morphology is highly significant as they determine the final geometrical shapes and pore architectures of the mesoporous silica nanoparticles. These shapes (spheres, oblong, platelets, short rods and long rods, and others) (Figure 1) and pore architectures (hexagonal, cubic, lamellar, and others) were recently reported to have a role in cellular responses. Differences in shapes also affect the biodistribution and clearance upon administration depending on the route of drug administration (Shao et al., 2017; Zheng et al., 2018). As dose uniformity is one of the required quality aspects for pharmaceuticals, spherical mesoporous nanoparticles (Leena et al., 2019) have been frequently researched as a potential delivery carrier for drugs owing to the ease of controlling the homogeneity of the drug loading efficiency as compared to other non-spherical shapes. However, Shao et al. (2017) found that differences in endocytosis routes caused long-rod-shaped MSN to exhibit more intracellular internalization than short-rod-shaped MSN and sphere-shaped MSN in both cancer and normal cells. In 2018, Zhang and co-workers researched on the oral delivery of indomethacin using MSN where it was found that rod-shaped MSN had higher drug loading (29.04%) and more enhanced dissolution compared to sphere-shaped MSN (22.29%) (Zheng et al., 2018).

## Control of Pore Size, Pore Volume, and Meso-Structural Pore Order

Pore size is a crucial determinant in mesoporous silica nanoparticles which influence drug loading capacity and drug release profile (Muñoz et al., 2003; Izquierdo-Barba et al., 2005). The selection of surfactant species significantly affects the pore size and meso-structural ordering of the particles. It was observed that larger pores of mesoporous silica resulted from a longer chain length of surfactant and smaller pores resulted from those with a shorter chain length (Yano and Fukushima, 2004; Ganguly et al., 2010). Moreover, it appears that an increase in the concentration of surfactant yielded higher pore volume while an increase in the concentration of catalysts produced higher pore diameter, larger particle size, and lower pore volume (Seo et al., 2014; Soto et al., 2016; Wu et al., 2016). The resultant porosity–morphology of mesoporous silica nanoparticles can also be adjusted by optimizing the temperature, acidity, and stirring time during the synthesis process.

Numerous surfactant types have been observed to significantly influence meso-structural ordering arrangement of the mesoporous silica nanoparticles. Earlier account reported that the CTAB surfactant concentration influenced the meso-structural arrangement of MSN (Beck et al., 1992). Similar to the solvent evaporation method to prepare PLGA micro/nanoparticles (Doolaaneaa et al., 2014) and other complex emulsion or nanoemulsion-based formulations (Yusof et al., 2014; Mohamed et al., 2022), a sufficient concentration of surfactant molecules shall be employed to produce a stable emulsion, as in the case of the silica–surfactant interface. Here, the micelles cannot be formed or self-assembled at very low surfactant concentration thus the resultant nanoparticles would be template-deficient, in contrast, a disordered structure was resulted when CTAB concentration was too high (Beck et al., 1992). Moreover, TEOS, as a provider of the silica species, was reported to influence the meso-architectural ordering of mesoporous silica nanoparticles. It is claimed that the lower amount may be insufficient to craft the mesoporous structure; however, a higher TEOS amount showed a disordered mesostructure (Akinjokun et al., 2016). Therefore, an ideal balance has to be reached between the different components used. Furthermore, meso-structural ordering was usually reported to occur in a diluted aqueous medium where the acidity of the medium also played a crucial role (Palmqvist, 2003).

A co-templating agent like N, N-dimethylhexadecylamine (DMHA) could also be the co-modulator in controlling the pore size owing to its effect in stabilizing the silica-surfactant template solution during the nanoparticle synthesis (Gu et al., 2013). Another variety, a wormhole-like arrangement of mesoporous silica nanoparticles, was demonstrated when analogs of CTAB, namely, cetyltrimethylammonium chloride (CTAC), were used as the pore-generating template. For the stellate-like arrangement of mesoporous silica nanoparticles, it was generated by varying the counter ion to a much larger tosylate ion (CTATOS) which consequently increased the pore radius (Möller and Bein, 2017).

## Advantages Leading to Applicability in Drug Delivery

Over the recent years, mesoporous silica nanoparticle has gained wide popularity owing to its desirable properties. This is mainly due to their unique properties which make it possible to engineer a near-perfect carrier as the researchers desired. Some of the distinctive properties include the ordered particle shape, particle size, pore shape, pore size, and high specific surface areas over volume. The possibility of tuning and modification during MPS synthesis is endless: their pores can be hexagonal, cubic, lamellar, et cetera; and their morphologies can be spheres, rods, discs, powders, et cetera. Their ability to endure surface modification and functionalization also charmed the researchers. Recently, the surface morphology of mesoporous silica was modified to look like a virus while the interaction of lipid membrane toward it was investigated in detail (Haffner et al., 2021).

In contrast to traditional porous silica materials, mesoporous silica exhibits exceptionally organized pore pattern. The regular and customizable pore size and nano-architectured matrix, the gating mechanism of the pore opening, and the convenient functionalization of the surface silanol group (Pandita et al., 2014; Han et al., 2018) have made it promising drug carriers. Various model drugs including macromolecules, namely DNA (Tao et al., 2014), RNA (Tao et al., 2014; Hanafi-Bojd et al., 2016; Möller et al., 2016), proteins (Deodhar et al., 2017), and small molecular weight drugs such as ibuprofen (Heikkil et al., 2007) and doxorubicin (Yuan et al., 2016); and most recently, co-delivery of doxorubicin and paclitaxel Yan et al. (2020) have been loaded into various designs of the mesoporous silica nanoparticles. The presence of porous channels within nanoparticle matrices provides relatively huge internal spatial for drugs to be placed and diffuse out according to the exposed dissolution media.

## Safety Aspects of Mesoporous Silica for Drug Delivery

Silica is a natural inorganic substance that exists abundantly in our environment, in contrast to other metal oxides like iron oxides and titanium oxides. It possesses greater biocompatibility with the human body compared to those oxides. Moreover, U.S. Food and Drug Administration (Title 21: Sec. 172.480, 2017) already declared the nontoxic and biocompatible nature of silica or silica dioxide. Hence, mesoporous silica nanoparticles are also considered safe to be used in pharmaceutical formulations where it enters the human body as potential drug carrier.

As a type of synthesized silica, the mesoporous range are thermally, hydrothermally, and hydrolytically stable with high surface areas (≥1000 m^2^/g) and >1600°C melting point (Sigma-Aldrich, 2018). Thus, this has made them an ideal carrier in adsorption, separation, and catalysis in the engineering field, biomedical field, and pharmaceutical where sterilization is needed especially in tissue engineering (Arcos and Vallet-Regí, 2010) or any implant-based drug delivery systems. Furthermore, similar properties are also beneficial in pharmaceutical applications notably as drug carriers that can enhance the stability of the active pharmaceutical ingredient (API) within their mesopore matrices (Laitinen et al., 2013; Antonino et al., 2019).

## Mesoporous Silica as the Next-Generation Drug Delivery System

### Current Status and Challenges of Drug Delivery Systems

Translation from the lab to clinical use for many drug delivery systems (DDSs) delivering macromolecules such as peptide drugs, antibody-drug conjugates, DNA- and RNA-based drugs indicated for diseases like diabetes, cancer, and neurotherapeutic pain still remain a challenge. Preserving the labile macromolecules against manufacturing conditions while ensuring the active form of folded peptide molecules finally reaches the targeted site of action at sufficient concentration are among the common factors that bog down the progress to clinical use. Delivery of cytotoxic drugs to treat solid tumors and metastatic are difficult in terms of targeting (Kashkooli et al., 2021) with some carriers exhibiting untoward premature release of drugs causing intolerable adverse effects such as bone marrow toxicity, which precluded their clinical use (Cazzamalli et al., 2017). Largely dependent on the route of drug administration, the aforementioned are due to insufficient stability of the DDS carriers *in vivo*, poor bioavailability and solubility of drugs, and lack of specificity by targeting ligand. Various nanoparticles including mesoporous silica-based, decorated, coated, cloaked, or linked with stimuli-responsive materials and some with targeting ligands have been synthesized to overcome the challenges (Wang et al., 2019; Yan et al., 2019; Hu et al., 2021; Zhang et al., 2021; Yang et al., 2022). Recently, an improved performance, targeted delivery of mesoporous organosilica nanoparticles (MONs) has been designed to be immunogenic by coating it with 4T1 breast cancer cell membrane while responsive to X-ray as the external activator that controlled the drug release (Shao et al., 2020). An orally delivered diselenide-bridged MON conjugated to polyethyleneimine demonstrated the ability to scavenge pro-inflammatory cell-free-DNA and reactive oxygen species (ROS) in ulcerative colitis and Crohn’s disease mouse model (Shi et al., 2022). In general, desirable characteristics of an NGDDS include non-toxic, biocompatible, having a robust synthetic pathway, tunable functionalization with any stimuli molecules or targeting ligands, customizable size, sufficient drug payload, ability to slowly release the drugs, and exhibit properties that can enhance solubility and permeability of the encapsulated drugs.

#### Improvement in Solubility and Permeability

The process of releasing the orally delivered drug from any dosage form to be absorbed into the human body system can be categorized as either immediate or modified drug release. Immediate drug release formulations dissolve within 30 min upon encountering bulk stomach solution, hence they are preferred when a fast onset of action is required during the therapy (Khaled et al., 2018; Sager et al., 2019). Unlike immediate-release, modified-release formulations can deliver a relatively more accurate dose of an active pharmaceutical ingredient (API) by controlling its release rate (delayed release, sustained release, prolonged release, et cetera) or by releasing at a specified targeted site in the body (H. Yan et al., 2020; Yang et al., 2018; Yan et al., 2020). By modifying the drug release, the therapeutic level of the drugs or any therapeutic compound can be achieved more effectively without the need for multiple dosing unlike the conventional formulations resulting in minimization of the anticipated side effects.

Mesoporous silica nanoparticles continue to show their great potential for oral drug delivery due to their large pore volumes, good stability, tunable particle, and pore shape and size as well as large surface area over volumes (Chen et al., 2004; Linbo et al., 2013). Such desirable properties generally lead to high drug loading during fabrication and improved solubility and dissolution rate upon oral consumption (Chaudhari and Gupte, 2017). The cumulative effect is a notable improvement in the bioavailability of the drugs in the blood owing to an increase in solubility and permeability properties. Carboxyl-functionalized MSN loaded with nimesulide and indomethacin exhibited an excellent *in vivo* bioavailability at 4–8 h with plasma concentration (C_max_ = 104.1 ± 5.4 μg/ml and C_max_ = 160.8 ± 8.1 μg/ml, respectively) along with effective anti-inflammatory effects (Gou et al., 2021). In addition to providing protection against harsh conditions in the gastrointestinal tract (GI tract), MSN was also found to be having good biocompatibility and low toxicity. There were no significant changes found for lipopolysaccharide in the serum and pathological characteristics after oral administration of mesoporous silica nanoparticles (Vol and Gribova, 2015).

In 2019, C. Han and co-workers (2019) synthesized carvedilol-loaded mesoporous silica and mesoporous carbon nanoparticles. They found that both carriers had low cytotoxicity level with viability of Caco-2 cells of more than 80% even for the highest sample concentration (500 μg/ml). The carbon carrier showed excellent drug loading efficiency of 16.7% while the silica carrier exhibited 7.9% loading (C. Han et al., 2019). The silica carrier exhibited a better dissolution profile with >70% cumulative dissolution, compared to the carbon carrier indicating better compatibility between the drug and carrier with little to no interaction resulting in more efficient drug release.

Mehmood et al. (2020) had researched the oral delivery of a hepatitis C virus inhibitor called sofosbuvir for sustained-release formulation in order to improve its bioavailability. Mesoporous silica nanoparticles were subjected to amino-functionalization using 3-aminopropyl triethoxysilane (APTES) and proceeded with drug loading. The formulation showed thermal stability while successfully entrapping 29.13% of the API. A significant increase in sofosbuvir bioavailability was observed up to 2-fold with a 3-fold prolonged-release behavior during *in-vivo* study using Sprague–Dawley rats.

#### Increase Bioavailability by Delayed-Release Strategy

The strong stability and advantageous features of mesoporous silica empowered its suitability to be used in the modified release formulations (Chen et al., 2004; Muhammad et al., 2011; Linbo et al., 2013). Oral delivery of the antibiotic meropenem was researched by Raza et al. (2021), where the researchers used liquid carbon dioxide to load the API into the mesoporous silica along with Eudragit® coating. The latter is responsible to provide the delayed-release property. The formulation was successfully able to exhibit enteric coating function where permeability was significantly increased to more than 2-fold for absorptive transport without any decrease in its antibacterial activity during the *in-vitro* antibacterial activity and time-kill assay against *S. aureus* and *P. aeruginosa.*

In 2020, Cai et al. (2020) designed a delayed-release oral dosage formulation of an anticancer drug (doxorubicin), for colon-targeted delivery using chitosan-gated, hollow mesoporous silica spheres. The formulation was found to have 35.2% drug loading while exhibiting a colonic enzyme-responsive manner of drug release during *in-vitro* study. These chitosan-capped mesoporous silica nanospheres were also biocompatible and stable according to the cell cytotoxicity results.

#### A Promising Carrier for Oral Delivery of Insulin

Oral insulin delivery has many issues due to the high possibility of degradation of the API (USP32–NF27, 2013). Research for insulin by oral delivery has been quite a while with none yet to enter the market. Mesoporous silica nanoparticles may be a promising NGDDS that researchers can focus on for the oral route. Formulation with mesoporous silica nanoparticles demonstrated the ability to protect insulin and improved its permeability without involving high temperature during the drug loading process (Siavashani et al., 2014; Agrawal et al., 2017). Mesoporous silica nanoparticles can also be modified with functionalization or coating as strategies to modify the drug release (Agrawal et al., 2017; Mahony and Morris, 2021). Gao et al. (2021) functionalized the mesoporous silica by coating it with deoxycholic acid and sulfobetaine-12 to load peptide drug, i.e., insulin. The zwitterionic coating appeared to be compatible with the mesoporous silica and resulted in a high drug loading of 22.2% with improved cellular permeability up to 8-fold for E12 cells and 10-fold for Caco-2 cells. Furthermore, the *in-vivo* study result was also outstanding whereby an effective hypoglycaemic effect was observed in diabetic rats.

## Conclusion

Based on the robust synthesis pathway that created various mesoporous morphology shape of the silica-based nanoparticles, this potential drug delivery carrier could be a very promising NGDDS. As the oral route is the most convenient for drug administration coupled with natural characteristics of mesoporous desirable for this route, and ease of scaling-up owing to established advancement that has been made in oral dosage form technology, it is recommended to delve into this innovative technology with more efforts focusing on oral drug delivery in the future.

## References

[B1] Agrawal G. R. Wakte P. Shelke S. (2017). Formulation, Physicochemical Characterization and *In Vitro* Evaluation of Human Insulin-Loaded Microspheres as Potential Oral Carrier. Prog. Biomater. 6 (3), 125–136. 10.1007/s40204-017-0072-z 28864917 PMC5597563

[B2] Akinjokun A. I. Ojumu T. V. Ogunfowokan A. O. (2016). Biomass, Abundant Resources for Synthesis of Mesoporous Silica Material. Microporous Mesoporous Mater. IntechOpen. 10.5772/63463

[B3] Antonino R. S. C. M. Q. Ruggiero M. Song Z. Nascimento T. L. Lima E. M. Bohr A. (2019). Impact of Drug Loading in Mesoporous Silica-Amorphous Formulations on the Physical Stability of Drugs with High Recrystallization Tendency. Int. J. Pharm. X 1, 100026–102590. 10.1016/j.ijpx.2019.100026 31517291 PMC6733286

[B4] Arcos D. Vallet-Regí M. (2010). Sol-Gel Silica-Based Biomaterials and Bone Tissue Regeneration. Acta Biomater. 6 (8), 2874–2888. 10.1016/j.actbio.2010.02.012 20152946

[B5] Beck J. S. Vartuli J. C. Roth W. J. Leonowicz M. E. Kresge C. T. Schmitt K. D. (1992). A New Family of Mesoporous Molecular Sieves Prepared with Liquid Crystal Templates. J. Am. Chem. Soc. 114 (27), 10834–10843. 10.1021/ja00053a020

[B6] Cai D. Han C. Liu C. Ma X. Qian J. Zhou J. (2020). Chitosan-Capped Enzyme-Responsive Hollow Mesoporous Silica Nanoplatforms for Colon-Specific Drug Delivery. Nanoscale Res. Lett. 15 (1), 123. 10.1186/s11671-020-03351-8 32488526 PMC7266918

[B73] Cazzamalli S. Corso A. D. Neri D. (2017). Targeted Delivery of Cytotoxic Drugs: Challenges, Opportunities and New Developments. Chimia (Aurau) 71 (10), 712–715. 10.2533/chimia.2017.712 PMC5844459 29070415

[B7] Chaudhari S. Gupte A. (2017). Mesoporous Silica as a Carrier for Amorphous Solid Dispersion. Bjpr 16 (6), 1–19. 10.9734/bjpr/2017/33553

[B8] Chen B.-C. Lin H.-P. Chao M.-C. Mou C.-Y. Tang C.-Y. (2004). Mesoporous Silica Platelets with Perpendicular Nanochannels via a Ternary Surfactant System. Adv. Mat. 16 (18), 1657–1661. 10.1002/adma.200306327

[B10] Deodhar G. V. Adams M. L. Trewyn B. G. (2017). Controlled Release and Intracellular Protein Delivery from Mesoporous Silica Nanoparticles. Biotechnol. J. 12 (1), 1600408. 10.1002/biot.201600408 27973750

[B11] Doolaanea A. A. Mansor N' Mohd Nor N. H. Mohamed F. (2014). Cellular Uptake of Nigella Sativa Oil-PLGA Microparticle by PC-12 Cell Line. J. Microencapsul. 31 (6), 600–608. 10.3109/02652048.2014.898709 24697178

[B12] Ganguly A. Ahmad T. Ganguli A. K. (2010). Silica Mesostructures: Control of Pore Size and Surface Area Using a Surfactant-Templated Hydrothermal Process. Langmuir 26 (18), 14901–14908. 10.1021/la102510c 20735023

[B13] Gao Y. He Y. Zhang H. Zhang Y. Gao T. Wang J. H. (2021). Zwitterion-Functionalized Mesoporous Silica Nanoparticles for Enhancing Oral Delivery of Protein Drugs by Overcoming Multiple Gastrointestinal Barriers. J. Colloid Interface Sci. 582, 364–375. 10.1016/j.jcis.2020.08.010 32861041

[B14] Gou K. Wang Y. Guo X. Wang Y. Bian Y. Zhao H. (2021). Carboxyl-Functionalized Mesoporous Silica Nanoparticles for the Controlled Delivery of Poorly Water-Soluble Non-Steroidal Anti-Inflammatory Drugs. Acta Biomater. 134, 576–592. 10.1016/j.actbio.2021.07.023 34280558

[B15] Gu J. Huang K. Zhu X. Li Y. Wei J. Zhao W. (2013). Sub-150 Nm Mesoporous Silica Nanoparticles with Tunable Pore Sizes and Well-Ordered Mesostructure for Protein Encapsulation. J. Colloid Interface Sci. 407, 236–242. 10.1016/j.jcis.2013.06.028 23866201

[B45] Haffner S. M. Parra-Ortiz E. Browning K. L. Jorgensen E. Skoda M. W. A. Montis C. (2021). Membrane Interactions of Virus-like Mesoporous Silica Nanoparticles. ACS Nanotechnology 15 (4), 6787–6800. 10.1021/acsnano.0c10378 33724786

[B16] Han C. Huang H. Dong Y. Sui X. Jian B. Zhu W. (2019). A Comparative Study of the Use of Mesoporous Carbon and Mesoporous Silica as Drug Carriers for Oral Delivery of the Water-Insoluble Drug Carvedilol. Molecules 24 (9), 1–14. 10.3390/molecules24091770 PMC6539599 31067732

[B17] Han Y. H. Kankala R. K. Wang S. B. Chen A. Z. (2018). Leveraging Engineering of Indocyanine Green-Encapsulated Polymeric Nanocomposites for Biomedical Applications. Nanomater. (Basel) 8 (6), 360. 10.3390/nano8060360 PMC6027497 29882932

[B18] Hanafi-Bojd M. Y. Ansari L. Malaekeh-Nikouei B. (2016). Codelivery of Anticancer Drugs and siRNA by Mesoporous Silica Nanoparticles. Ther. Deliv. 7 (9), 649–655. 10.4155/tde-2016-0045 27582236

[B19] Hao N. Li L. Zhang Q. Huang X. Meng X. Zhang Y. (2012). The Shape Effect of PEGylated Mesoporous Silica Nanoparticles on Cellular Uptake Pathway in Hela Cells. Microporous Mesoporous Mater. 162, 14–23. 10.1016/j.micromeso.2012.05.040

[B20] Heikkilä T. Salonen J. Tuura J. Hamdy M. S. Mul G. Kumar N. (2007). Mesoporous Silica Material TUD-1 as a Drug Delivery System. Int. J. Pharm. 331, 133–138. 10.1016/j.ijpharm.2006.09.019 17046183

[B21] Hu H. Yang C. Zhang F. Li M. Tu Z. Mu L. (2021). A Versatile and Robust Platform for the Scalable Manufacture of Biomimetic Nanovaccines. Adv. Sci. (Weinh) 8, 2002020. 10.1002/advs.202002020 34386315 PMC8336609

[B22] Huang X. Teng X. Chen D. Tang F. He J. (2010). The Effect of the Shape of Mesoporous Silica Nanoparticles on Cellular Uptake and Cell Function. Biomaterials 31 (3), 438–448. 10.1016/j.biomaterials.2009.09.060 19800115

[B23] Inagaki S. Koiwai A. Suzuki N. Fukushima Y. Kuroda K. (1996). Syntheses of Highly Ordered Mesoporous Materials, FSM-16, Derived from Kanemite. Bcsj 69, 1449–1457. 10.1246/bcsj.69.1449

[B24] Izquierdo-Barba I. Martinez A. Doadrio A. L. Pérez-Pariente J. Vallet-Regí M. (2005). Release Evaluation of Drugs from Ordered Three-Dimensional Silica Structures. Eur. J. Pharm. Sci. 26 (5), 365–373. 10.1016/j.ejps.2005.06.009 16185852

[B25] Johansson E. M. Ballem M. A. Córdoba J. M. Odén M. (2011). Rapid Synthesis of SBA-15 Rods with Variable Lengths, Widths, and Tunable Large Pores. Langmuir 27 (8), 4994–4999. 10.1021/la104864d 21413751

[B26] Khaled S. A. Alexander M. R. Wildman R. D. Wallace M. J. Sharpe S. Yoo J. (2018). 3D Extrusion Printing of High Drug Loading Immediate Release Paracetamol Tablets. Int. J. Pharm. 538 (1-2), 223–230. 10.1016/j.ijpharm.2018.01.024 29353082

[B27] Kim J. M. Stucky G. D. (2000). Synthesis of Highly Ordered Mesoporous Silica Materials Using Sodium Silicate and Amphiphilic Block Copolymers. Chem. Commun. 13, 1159–1160. 10.1039/b002362k

[B28] Laitinen R. Löbmann K. Strachan C. J. Grohganz H. Rades T. (2013). Emerging Trends in the Stabilization of Amorphous Drugs. Int. J. Pharm. 453 (1), 65–79. 10.1016/j.ijpharm.2012.04.066 22569230

[B29] Lin X. Yang H. Su L. Yang Z. Tang X. (2018). Effect of Size on the *In Vitro*/*In Vivo* Drug Release and Degradation of Exenatide-Loaded PLGA Microspheres. J. Drug Deliv. Sci. Technol. 45, 346–356. 10.1016/j.jddst.2018.03.024

[B30] Linbo H. Zhou Y. He T. Song G. Wu F. Jiang F. (2013). One-Pot Morphology-Controlled Synthesis of Various Shaped Mesoporous Silica Nanoparticles. J. Mater. Sci. 48 (17), 5718–5726. 10.1007/s10853-013-7501-8

[B31] Madaan K. Kumar S. Poonia N. Lather V. Pandita D. (2014). Dendrimers in Drug Delivery and Targeting: Drug-Dendrimer Interactions and Toxicity Issues. J. Pharm. Bioallied Sci. 6 (3), 139–150. 10.4103/0975-7406.130965 25035633 PMC4097927

[B32] Mahony T. F. O. Morris M. A. (2021). Hydroxylation Methods for Mesoporous Silica and Their Impact on Surface Functionalisation. Microporous Mesoporous Mater. 317, 1387–1811. 10.1016/j.micromeso.2021.110989

[B33] Mehmood Y. Khan I. U. Shahzad Y. Khan R. U. Khalid S. H. Yousaf A. M. (2020). Amino-Decorated Mesoporous Silica Nanoparticles for Controlled Sofosbuvir Delivery. Eur. J. Pharm. Sci. 143, 105184. 10.1016/j.ejps.2019.105184 31846695

[B34] Mohamed F. Hamidon N. E. Adina A. B. Ardini Y. D. Mohd-Shafri M. A. (2022). Fabrication, Physicochemical and Rheological Characterisation of a Drug-Therapeutic Oils (Doxycycline Hyclate-Nigella Sativa-Eugenol) Complex Emulsion Stabilised by Lecithin and Hydroxypropyl Methylcellulose Intended for Delivery into Periodontal Pocket. Malays. J. Med. Health Sci. 18 (1), 20–28.

[B35] Möller K. Bein T. (2017). Talented Mesoporous Silica Nanoparticles. Chem. Mater. 29 (1), 371–388. 10.1021/acs.chemmater.6b03629

[B36] Möller K. Müller K. Engelke H. Bräuchle C. Wagner E. Bein T. (2016). Highly Efficient siRNA Delivery from Core-Shell Mesoporous Silica Nanoparticles with Multifunctional Polymer Caps. Nanoscale 8 (7), 4007–4019. 10.1039/c5nr06246b 26819069

[B37] Moradi Kashkooli F. Soltani M. Momeni M. M. Rahmim A. (2021). Enhanced Drug Delivery to Solid Tumors via Drug-Loaded Nanocarriers: An Image-Based Computational Framework. Front. Oncol. 11, 655781. 10.3389/fonc.2021.655781 34249692 PMC8264267

[B38] Muhammad F. Guo M. Qi W. Sun F. Wang A. Guo Y. (2011). pH-Triggered Controlled Drug Release from Mesoporous Silica Nanoparticles via Intracelluar Dissolution of ZnO Nanolids. J. Am. Chem. Soc. 133 (23), 8778–8781. 10.1021/ja200328s 21574653

[B39] Muñoz B. Rámila A. Pérez-Pariente J. Díaz I. Vallet-Regí M. (2003). MCM-41 Organic Modification as Drug Delivery Rate Regulator. Chem. Mater. 15 (2), 500–503. 10.1021/cm021217q

[B40] Nandiyanto A. B. D. Kim S.-G. Iskandar F. Okuyama K. (2009). Synthesis of Spherical Mesoporous Silica Nanoparticles with Nanometer-Size Controllable Pores and Outer Diameters. Microporous Mesoporous Mater. 120, 447–453. 10.1016/j.micromeso.2008.12.019

[B41] Narayan R. Nayak U. Y. Raichur A. M. Garg S. (2018). Mesoporous Silica Nanoparticles: A Comprehensive Review on Synthesis and Recent Advances. Pharmaceutics 10, 118. 10.3390/pharmaceutics10030118 PMC6160987 30082647

[B42] Palmqvist A. E. C. (2003). Synthesis of Ordered Mesoporous Materials Using Surfactant Liquid Crystals or Micellar Solutions. Curr. Opin. Colloid & Interface Sci. 8 (2), 145–155. 10.1016/s1359-0294(03)00020-7

[B43] Patil L. D. Verma U. Patil U. D. Naik J. B. Narkhede J. S. (2019). Inclusion of Aceclofenac in Mesoporous Silica Nanoparticles: Drug Release Study and Statistical Optimization of Encapsulation Efficiency by Response Surface Methodology. Mater. Technol. 34, 751–763. 10.1080/10667857.2019.1624301

[B44] Pevzner S. Regev O. Yerushalmi-Rozen R. (1999). Thin Films of Mesoporous Silica: Preparation and Characterization. Curr. Opin. Colloid & Interface Sci. 4 (6), 420–427. 10.1016/s1359-0294(00)00018-2

[B46] Raza A. Sime F. B. Cabot P. J. Roberts J. A. Falconer J. R. Kumeria T. (2021). Liquid CO_2_ Formulated Mesoporous Silica Nanoparticles for pH-Responsive Oral Delivery of Meropenem. ACS Biomater. Sci. Eng. 7 (5), 1836–1853. 10.1021/acsbiomaterials.0c01284 33438994

[B47] Sager M. Grimm M. Jedamzik P. Merdivan S. Kromrey M. L. Hasan M. (2019). Combined Application of MRI and the Salivary Tracer Technique to Determine the *In Vivo* Disintegration Time of Immediate Release Formulation Administered to Healthy, Fasted Subjects. Mol. Pharm. 16 (4), 1782–1786. 10.1021/acs.molpharmaceut.8b01320 30821987

[B49] Seo J. W. Lee W. Nam S. Ryoo H. Kim J-M. Ko C. H. (2014). Mesoporous Structure Control of Silica in Room-Temperature Synthesis Under Basic Conditions. J. Nanomater., 149654. 10.1155/2015/149654

[B50] Shao D. Lu M. M. Zhao Y. W. Zhang F. Tan Y. F. Zheng X. (2017). The Shape Effect of Magnetic Mesoporous Silica Nanoparticles on Endocytosis, Biocompatibility and Biodistribution. Acta Biomater. 49, 531–540. 10.1016/j.actbio.2016.11.007 27836804

[B51] Shao D. Zhang F. Chen F. Zheng X. Hu H. Yang C. (2020). Biomimetic Diselenide-Bridged Mesoporous Organosilica Nanoparticles as an X-Ray-Responsive Biodegradable Carrier for Chemo-Immunotherapy. Adv. Mater 32 (50), e2004385. 10.1002/adma.202004385 33164250

[B52] Shi C. Dawulieti J. Shi F. Yang C. Qin Q. Shi T. (2022). A Nanoparticulate Dual Scavenger for Targeted Therapy of Inflammatory Bowel Disease. Sci. Adv. 8 (4), eabj2372. 10.1126/sciadv.abj2372 35089791 PMC8797786

[B53] Siavashani A. Z. Nazarpak M. H. Bakhsh F. F. Toliyat T. Solati-Hashjin M. (2014). Preparation of Mesoporous Silica Nanoparticles for Insulin Drug Delivery. Adv. Mater. Res. 829, 251–257. 10.4028/www.scientific.net/amr.829.251

[B74] Sigma-Aldrich (2018). Mesitylene. Available at: https://www.sigmaaldrich.com/MY/en/product/sial/m7200

[B54] Soto R. J. Yang L. Schoenfisch M. H. (2016). Functionalized Mesoporous Silica via an Aminosilane Surfactant Ion Exchange Reaction: Controlled Scaffold Design and Nitric Oxide Release. ACS Appl. Mater Interfaces 8 (3), 2220–2231. 10.1021/acsami.5b10942 26717238 PMC4734612

[B55] Tao C. Zhu Y. Xu Y. Zhu M. Morita H. Hanagata N. (2014). Mesoporous Silica Nanoparticles for Enhancing the Delivery Efficiency of Immunostimulatory DNA Drugs. Dalton Trans. 43 (13), 5142–5150. 10.1039/c3dt53433b 24496286

[B56] USP32–NF27 (2013). Insulin Human. Available at http://www.uspbpep.com/usp32/pub/data/v32270/usp32nf27s0_m40600.html (Retrieved on September 22, 2021).

[B57] Vol A. Gribova O. (2015). Methods and Compositions for Oral Administration of Protein and Peptide Therapeutic Agents (Patent No. 8936786). Available at https://patents.google.com/patent/US8936786B2/en?oq=8936786.

[B58] Wang Y. Zhang F. Wang Y. Ren J. Li C. Liu X. (2009). Synthesis of Length Controllable Mesoporous SBA-15 Rods. Mater. Chem. Phys. 115, 649–655. 10.1016/j.matchemphys.2009.01.027

[B59] Wang Z. Zhang F. Shao D. Chang Z. Wang L. Hu H. (2019). Janus Nanobullets Combine Photodynamic Therapy and Magnetic Hyperthermia to Potentiate Synergetic Anti‐Metastatic Immunotherapy. Adv. Sci. 6, 1901690. 10.1002/advs.201901690 PMC6864517 31763151

[B60] Wu L. Jiao Z. Wu M. Song T. Zhang H. (2016). Formation of Mesoporous Silica Nanoparticles with Tunable Pore Structure as Promising Nanoreactor and Drug Delivery Vehicle. RSC Adv. 6 (16), 13303–13311. 10.1039/c5ra27422b

[B61] Yan H. Chen X. Bao C. Yi J. Lei M. Ke C. (2020). Synthesis and Assessment of CTAB and NPE Modified Organo-Montmorillonite for the Fabrication of Organo-Montmorillonite/Alginate Based Hydrophobic Pharmaceutical Controlled-Release Formulation. Colloids Surf. B Biointerfaces 191, 110983. 10.1016/j.colsurfb.2020.110983 32208326

[B62] Yan J. Xu X. Zhou J. Liu C. Zhang L. Wang D. (2020). Fabrication of a pH/Redox-Triggered Mesoporous Silica-Based Nanoparticle with Microfluidics for Anticancer Drugs Doxorubicin and Paclitaxel Codelivery. ACS Appl. Bio Mater. 3, 1216–1225. 10.1021/acsabm.9b01111 35019322

[B63] Yan X. Meng J. Hu X. Feng R. Zhou M. (2019). Synthesis of Thiol-Functionalized Mesoporous Silica Nanoparticles for Adsorption of Hg^2+^ from Aqueous Solution. J. Sol-Gel Sci. Technol. 89 (3), 617–622. 10.1007/s10971-019-04923-6

[B64] Yang D. Luo W. Wang J. Zheng M. Liao X. H. Zhang N. (2018). A Novel Controlled Release Formulation of the Pin1 Inhibitor ATRA to Improve Liver Cancer Therapy by Simultaneously Blocking Multiple Cancer Pathways. J. Control Release 269, 405–422. 10.1016/j.jconrel.2017.11.031 29170140 PMC6290999

[B65] Yang Y. Chen F. Xu N. Yao Q. Wang R. Xie X. (2022). Red-Light-Triggered Self-Destructive Mesoporous Silica Nanoparticles for Cascade-Amplifying Chemo-Photodynamic Therapy Favoring Antitumor Immune Responses. Biomaterials 281, 121368. 10.1016/j.biomaterials.2022.121368 35030436

[B66] Yano K. Fukushima Y. (2004). Synthesis of Mono-Dispersed Mesoporous Silica Spheres with Highly Ordered Hexagonal Regularity Using Conventional Alkyltrimethylammonium Halide as a Surfactant Electronic Supplementary Information (ESI) Available: Time Courses of Particle Size and Scattering Intensity of Samples Obtained with TEOS and C16TMACl. See http://www.rsc.Org/suppdata/jm/b3/b313712k/. J. Mat. Chem. 14 (10), 1579–1584. 10.1039/b313712k

[B67] Yuan Z. Pan Y. Cheng R. Sheng L. Wu W. Pan G. (2016). Doxorubicin-Loaded Mesoporous Silica Nanoparticle Composite Nanofibers for Long-Term Adjustments of Tumor Apoptosis. Nanotechnology 27 (24), 245101. 10.1088/0957-4484/27/24/245101 27172065

[B68] Yusof F. A. Mohd-Shafri M. A. Yaakob K. I. Mohamed F. (2014). Formulation and Stability Testing of Gentamicin-*N. Sativa* Fusion Emulsions for Osteo-Healing Application. Int. J. Pharm. Pharm. Sci. 6 (11), 171–176.

[B69] Zhang F. Chen F. Yang C. Wang L. Hu H. Li X. (2021). Coordination and Redox Dual-Responsive Mesoporous Organosilica Nanoparticles Amplify Immunogenic Cell Death for Cancer Chemoimmunotherapy. Small 17 (26), 2170130. 10.1002/smll.202170130 34081391

[B70] Zhang W. Zheng N. Chen L. Xie L. Cui M. Li S. (2018). Effect of Shape on Mesoporous Silica Nanoparticles for Oral Delivery of Indomethacin. Pharmaceutics 11 (1), 4. 10.3390/pharmaceutics11010004 PMC6359657 30583601

[B71] Zhao D. Huo Q. Feng J. Chmelka B. F. Stucky G. D. (1998). Nonionic Triblock and Star Diblock Copolymer and Oligomeric Surfactant Syntheses of Highly Ordered, Hydrothermally Stable, Mesoporous Silica Structures. J. Am. Chem. Soc. 120, 6024–6036. 10.1021/ja974025i

[B72] Zheng N. Li J. Xu C. Xu L. Li S. Xu L. (2018). Mesoporous Silica Nanorods for Improved Oral Drug Absorption. Artif. Cells Nanomed. Biotechnol. 46 (6), 1132–1140. 10.1080/21691401.2017.1362414 28783976

